# Effects of lipopolysaccharide administration on thymus damage, antioxidant capacity and immune function in weaned piglets

**DOI:** 10.2478/jvetres-2025-0018

**Published:** 2025-03-25

**Authors:** Lingna Bai, Yijie Jiang, Xi Li, Wanting Yu, Wenlu Zhu, Guotong Zhao, Tingyu Yang, Yunxiao Zhou, Jinyan Li, Yong Li

**Affiliations:** College of Animal Science and Technology, Jiangxi Agricultural University, 330045 Nanchang, Jiangxi, China; Agricultural Technology Extension Center of Pingxiang City, 337000 Pingxiang, Jiangxi, China

**Keywords:** lipopolysaccharide, piglet, thymus, inflammation, antioxidant capacity

## Abstract

**Introduction:**

Piglets are vulnerable to stress during weaning because of changes in the feeding environment, nutrients, and other growth-impacting conditions. In this study, stress injury was modelled by continuous intraperitoneal injection of lipopolysaccharide (LPS) and was used to investigate the dynamics of antioxidant indices and immunoinflammatory factors in the piglet thymus.

**Material and Methods:**

Forty-eight weaned piglets were divided into an LPS group and a control group. One group was injected with LPS solution (100 μg/kg) and the other with sterile saline daily. The experiment ran over 13 days, and six piglets from each group were euthanised for necropsy on days 1, 5, 9 and 13. Thymic tissues were collected, and the antioxidant indices and mRNA expression levels of related genes were measured by enzyme activity assay and reverse-transcription quantitative PCR.

**Results:**

In the LPS group, catalase activities were significantly increased on days 1 and 5, that of superoxide dismutase was significantly higher on day 9 and glutathione activity was elevated throughout. Messenger RNA (mRNA) expression of the toll-like receptor 4 (TLR4) pathway, interleukin (IL) 6, and IL-2 increased in the thymus on day 1. By day 5, the mRNA expression of the TLR pathway, the janus kinase (JAK)/signal transducer and activator of transcription (STAT) pathway, the kelch-like ECH-associated protein 1 (Keap1)/nuclear factor erythroid 2-related factor 2 (Nrf2) pathway, tumour necrosis factor α, IL-10, IL-6 and IL-2 were decreased. On day 13, the mRNA expression levels of the TLR4 and Keap1/Nrf2 pathways, TNF-α, IL-10 and IL-6 increased again.

**Conclusion:**

Continuous LPS induction led to high activation of the thymic immune system in piglets during the prophase. However, this activation was accompanied by atrophy and immunosuppression mid-experiment. Nevertheless, the immune function gradually recovered in the later stages.

## Introduction

Lipopolysaccharide (LPS) is a component of the cell walls of Gram-negative bacteria and is their primary virulence factor. It is released primarily during bacterial cell death or lysis (22). Lipopolysaccharide is comprised of two distinct regions: hydrophobic lipid A and hydrophilic long-chain polysaccharides. The polysaccharide structure can be further divided into core polysaccharides and specific O-antigens. Upon entering the organism, lipopolysaccharide binds to LPS-binding protein (LBP), forming a complex designated LPS-LBP. The complex is then presented to the CD14 of target cells, which further forms the LPS-LBP-CD14 triplex and translocates to the toll-like receptor 4 (TLR4)-myeloid differentiation protein-2 protein complex (44). This marks the completion of the recognition process for LPS (31). The toll-interleukin-1 receptor domain (TIR) of TLR4 interacts with the TIR domain of myeloid differentiation factor 88 (MyD88), leading to the interaction of MyD88’s N-terminal death domain (DD) with factors possessing homotypic DD domains such as interleukin-1 receptor–associated kinase. This leads to the phosphorylation and subsequent release of MyD88 into the cytoplasm (49). Subsequently, nuclear factor (NF) kappa-B (κB) is activated and translocated to the nucleus for transcription. It acts as a major transcription factor, encoding downstream inflammatory mediators such as tumour necrosis factor α (TNF-α) and interleukin (IL) 6, thereby eliciting an inflammatory response (21). Lipopolysaccharide stimulates and activates the immune system (5), and there is notable elevation in the levels of various inflammatory mediators, including IL-1, interferon-gamma (IFN-γ), IL-6, and TNF-α (13). Yuan *et al*. (47) induced colitis in rats by intraperitoneal LPS injection and found that the messenger (m)RNA expression in IL-1β and TNF-α was upregulated throughout the colonic tissue. Zheng *et al*. (45) showed that intraperitoneal LPS administration in rats successfully induced cardiac dysfunction and an expression increase of the proinflammatory cytokines TNF-α, IL-1β, and IL-6 mRNA content and protein in serum.

The thymus is an essential central immune organ in piglets before sexual maturity. It is the site of T lymphocyte differentiation, development and maturation and secretes thymic peptides. In the early development of piglets, the thymus may atrophy and degenerate because of weaning stress, malnutrition and microbial infections (43). Thymic atrophy leads to a decrease in the number of thymocytes, initial T cells and output T-cells (29), which results in a decline in the host immunity, a weakened defence against disease and susceptibility to pathogens. Lipopolysaccharide induction results in decreased thymic mass, a decline in thymic DN and DP numbers and an impairment of thymic functionality (18, 28, 35). After LPS induction, the thymus may atrophy under stress, inducing tissue destruction and immunosuppression, but is able to restore its immune function through its own compensatory mechanisms (3). The expression of inflammatory factors, chemokines and cytokines is significantly upregulated during thymic atrophy, where IL-6 acts as a pivotal cytokine in the pathogenesis of this process (46). When inflammatory factors are overproduced, then an immune stress response in animals is induced, leading to damage to immune tissues and organs, which affects the health and growth of animals (34).

Lipopolysaccharide activates the immune and antioxidant systems of animals, inducing their responses to immune stress and oxidative stress. This causes the organism to secrete inflammatory factors such as TNF-α, IL-1 and IL-6, which result in an elevation of host temperature and a substantial expenditure of energy, leading to tissue damage in immune organs (40). Nitric oxide (NO) is a significant free radical that is capable of regulating a multitude of physiological functions and serves as a signalling molecule that modulates the immune response (1). The induction of inducible nitric oxide synthase (iNOS) in tissues can result in the sustained production of high concentrations of NO, which serves as an important inflammatory mediator and regulates the immune response process. However, its excessive secretion is also a significant contributor to tissue damage (11). Inducible NOS is regulated by a number of transcriptional pathways, including the NFκB pathway, the janus kinase/signal transducer and activator of transcription 1 (JAK/STAT-1) pathway and the activator protein-1 (AP-1) pathway. These pathways are dependent on the type of iNOS inducer (39, 51). Additionally, NO mitigates the inflammatory response by reducing the activity of NF-κB and TNF-α (12).

Animals possess intricate antioxidant defence mechanisms that enable them to overcome the challenges posed by oxidative stress. These mechanisms encompass three primary categories: endogenous anti-oxidative stress pathways, enzymatic antioxidant systems and non-enzymatic antioxidant systems (32). These systems function synergistically to effectively scavenge free radicals and repair oxidative damage, thereby maintaining redox balance. Enzymatic antioxidant systems contain superoxide dismutase (SOD), catalase (CAT) and glutathione peroxidase (GPH-Px), which catalyse the decomposition and transformation of free radicals, thereby reducing their toxicity (2). Non-enzymatic antioxidant systems include vitamin C, vitamin E, glutathione (GSH), carotenoids and the trace elements selenium and zinc. These compounds are capable of binding directly to free radicals, thereby neutralising their activity and reducing oxidative damage (38). The endogenous anti-oxidative stress pathway is the first line of defence against oxidative stress, which activates the expression of antioxidant genes through a series of signal transduction pathways to enhance the antioxidant capacity of cells. Among them, the nuclear factor erythroid 2-related factor 2 (Nrf2)/antioxidant response element (ARE) pathway is an important endogenous anti-oxidative stress pathway: when the organism is exposed to excessive reactive oxygen species (ROS), the ARE in the regulatory region upstream of the gene regulates the host production of a series of protective proteins, thereby mitigating cellular damage (14). As an activator of ARE (25), Nrf2 is a master cellular sensor of oxidative stress that binds to kelch-like ECH-associated protein 1 (Keap1) and is located in the cytoplasm (41). In typical circumstances, Nrf2 binds to its inhibitor Keap1, resulting in relatively level activity. However, when the cell is subjected to oxidative stress, the active site of Keap1 is oxidised, preventing Keap1 from binding to Nrf2 and consequently leading to the nuclear translocation of Nrf2 (20). Once ROS and reactive nitrogen species (RNS) exceed safe values, Nrf2 immediately induces ARE gene action to rapidly remove excessive ROS and RNS (19). Activation of the Nrf2 signalling pathway inhibits oxidative stress, thereby suppressing the expression of pro-apoptotic and pro-inflammatory genes regulated by NFκB, and attenuates the inflammatory response (36).

Gram-negative bacteria commonly occur in the feeding environment; which implies that LPS might become a principal virulence factor after Gram-negative bacteria invade piglets. The present study investigated the damage to and antioxidant capacity and immune capacity of the piglet’s thymus in response to LPS induction. This investigation provides more theoretical knowledge fundamental to understanding stress-related diseases in piglets.

## Material and Methods

### Animals and experimental design

Forty-eight 28-day-old clinically healthy weaned piglets (Duroc × Landrace × Large White with body weight of 6.65 ± 1.19 kg) were purchased from Jiangxi Aoyun Agricultural Development Co. (Jiangxi, China) and kept in sterilised experimental animal houses at Jiangxi Agricultural University. Following a day of acclimatisation to the new rearing environment, the piglets were acclimated for three days, during which they were fed and watered liberally. The piglets were identified by an ear tag and number and randomly divided into two equally sized groups: the experimental group (LPS group) and the control group (CON group). The experiment lasted for 13 days, during which piglets in the LPS group were injected intraperitoneally with LPS solution (100 μg/kg) daily, and those in the CON group were injected intraperitoneally with saline equal to the volume of LPS daily. Six piglets per group were randomly euthanised and necropsied on each of days 1, 5, 9 and 13, and the thymus was collected, quickly placed in liquid nitrogen and stored in a refrigerator at -80°C for subsequent analyses. During the experimental period, all procedures were approved by the Animal Care and Use Committee of Jiangxi Agricultural University (approval No. JXAULL-2022006 of June 17, 2022).

### Antioxidant evaluation

A 200 mg sample of thymus tissue was weighed and then homogenised in 1.8 % saline solution in order to produce a 10% homogenate. The homogenate was first subjected to protein concentration determination (BCA Protein Concentration Determination Kit; Wuhan Servicebio Technology Co., Hubei, China) and subsequently standardised to a consistent initial concentration. The homogenate was diluted to appropriate concentrations for the antioxidant enzyme kits used, of which there were six: for total antioxidant capacity (T-AOC; Cat. No. A015-1), superoxide dismutase (SOD; Cat. No. A001-3), catalase (CAT; Cat. No. A007-11), glutathione peroxidase (GSH-Px; Cat. No. A005), malondialdehyde (MDA; Cat. No. A003-1) and nitric oxide (NO; Cat. No. A013-2-1). The above kits were purchased from Nanjing Jiancheng Bioengineering Institute Co., (Nanjing, Jiangsu, China), and the experimental procedures were strictly in accordance with the instructions of the respective kits.

### Total RNA isolation and RT-qPCR

Total RNA was extracted from thymus tissues with Trizol reagent (Wuhan Servicebio Technology, Hubei, China), and its concentration and purity were detected with an ultraviolet spectrophotometer and adjusted to 1 μg/μL. Subsequently, mRNA was transcribed into complimentary DNA (cDNA) using StarScriptIII All-in-One RT Mix with gDNA Remover (GeneStar, Beijing, China) according to the instructions. A quantitative reverse-transcription PCR was performed on an ABI Quant Studio5 Flex PCR instrument (Applied Biosystems, Foster City, CA, USA) using PerfectStar Green qPCR Super Mix (Transgen Biotechnology, Beijing, China).

The total volume of PCR was 20 μL (1 μL of cDNA, 0.4 μL of each of the forward primer (10 μM) and reverse primer (10 μM), 10 μL of 2× PerfectStar Green qPCR Super Mix, 0.4 μL of passive reference dye (50×), and 7.8 μL of nuclease-free water). The amplification process was as follows: 94°C for 30 s, followed by 42 cycles of 94°C for 5 s and 60°C for 30 s. All primers were synthesised by Sangon Biotech Co. (Shanghai, China) and were as shown in Table 1. Glyceraldehyde 3-phosphate dehydrogenase was used as an internal reference, and the relative expression of the target gene was calculated according to the 2^-ΔΔCt^ formula.

**Table 1.** Primers used in RT-qPCR analysis

Gene	Primer sequence (5′-3′)	Product size (base pairs)	GenBank accession No.
GAPDH	F: CCTGGAGAAACCTGCAAAATA R: AACCTGGTCCTCAGTGTAGCC	100	NM_001206359.1
TLR4	F: GACGAAGACTGGGTGAGGAATGAAC R: CCTGGATGATGTTAGCAGCGATGG	124	NM_001113039.2
MyD88	F: CGTCTGGTCCATTGCTAGAACTC R: TTCTGATGGGCACCTGGAGAGAG	141	NM_001099923.1
NF-κB	F: CTGAGGCTATAACTCGCTTGGTGAC R: CATGTCCGCAATGGAGGAGAAGTC	131	NM_001114281.1
TNF-α	F: TCTATTTTGGGATCATTGCCC R: CCAGCCCCTCATTCTCTTTCT	127	NM_214022.1
IL-2	F: ACTTTCCAGGATGCTCAC R: ACTTCCTCCAGAGGTTTG	102	DQ852342.1
IL-6	F: ATAAGGGAAATGTCGAGGCTGTGC R: GGGTGGTGGCTTTGTCTGGATTC	93	NM_001252429.1
IL-10	F: GCATCCACTTCCCAACCA R: GCAACAAGTCGCCCATCT	108	NM_214041.1
JAK1	F: GACCGTCACCTGCTTTGAGA R: ACGAAGCTGATGTTGTCCGT	104	AB00601
STAT3	F: AGCCTCTCCGCAGAGTTCAA R: GCCCCCGTTCCCACAT	60	NM_001044580.1
iNOS	F: CAGCGGGATGACTTTCCAA R: TTGCAAGCAAGATCCCCTTT	60	NM_001143690.1
Keap1	F: ACGACGTGGAGACAGAAACGT R: GCTTCGCCGATGCTTCA	56	NM_001114671.1
Nrf2	F: GCCCCTGGAAGCGTTAAAC R: GGACTGTATCCCCAGAAGGTTGT	67	XM_003133500.5

1 GAPDH – glyceraldehyde 3-phosphate dehydrogenase; TLR4 – toll-like receptor 4; MyD88 – myeloid differentiation factor 88; NFκB – nuclear factor kappa B; TNF-α – tumour necrosis factor α; IL – interleukin; JAK1 – janus kinase 1; STAT3 – signal transducer and activator of transcription 3; iNOS – inducible nitric oxide synthase; Keap1 – kelch-like ECH-associated protein 1; Nrf2 – nuclear factor erythroid 2-related factor 2; F – forward; R – reverse

### Statistical analysis

Data were analysed by one way analysis of variance using IBM SPSS Statistics 25.0 software (IBM, Armonk, NY, USA) to compare the differences between the LPS and CON groups. A P-value < 0.05 was considered significant and one < 0.01 highly significant. All data was expressed as mean ± standard deviation and subsequently plotted using GraphPad Prism 8.0 software (GraphPad Software, San Diego, CA, USA).

## Results

### Effect of LPS induction on antioxidant parameters and NO indices in the thymus of weaned piglets

**Fig. 1.**
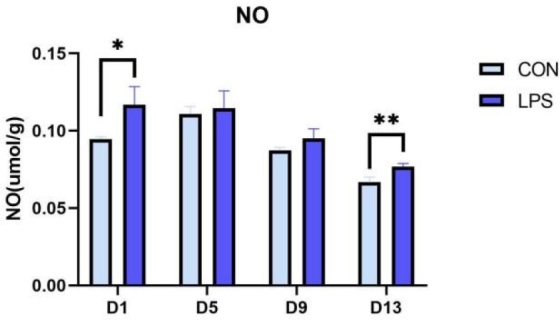
The nitric oxide (NO) secretion in the thymus of weaned piglets. CON – control group; LPS – lipopolysaccharide group; * – significant increase (P-value < 0.05); ** – highly significant increase (P-value < 0.01)

The antioxidant parameters in the thymus of piglets are shown in Table 2. During the induction by LPS, the activities of CAT, SOD and GSH-Px in the thymus of the LPS group were significantly elevated on different days in comparison with these activities in the CON group. Elevated activities of CAT were observed on days 1 and 5 (day 1 P-value < 0.01 and day 5 P-value < 0.05), and higher SOD activity was observed on day 9 (P-value < 0.05). Glutathione peroxidase activity was elevated throughout the experimental phase (days 1, 5 and 9 P-value < 0.05 and day 13 P-value < 0.01), while the activity of T-AOC and the content of MDA did not change significantly (P-value > 0.05). Thymic NO content, which is shown in Fig. 1, increased significantly on day 1 and day 13 (day 1 P-value < 0.05 and day 13 P-value < 0.01), but did not change significantly on days 5 and 9 (P-value > 0.05) compared with the CON group.

**Table 2.** The antioxidant parameters in the thymus of weaned piglets

	Group	Day 1	Day 5	Day 9	Day 13
T-AOC (mmol/g protein)	CON	0.1462 ± 0.00640	0.1077 ± 0.02364	0.0755 ± 0.01391	0.1027 ± 0.00941
	LPS	0.1527 ± 0.01974	0.1189 ± 0.00409	0.0787 ± 0.00503	0.0960 ± 0.00266
CAT (U/mg protein)	CON	1.3091 ± 0.12010	1.0701 ± 0.09588	1.1309 ± 0.04572	0.5535 ± 0.08402
	LPS	2.2744 ± 0.16687**	1.3503 ± 0.14927*	1.0992 ± 0.16026	0.6420 ± 0.09374
SOD (U/mg protein)	CON	15.4502 ± 1.37575	14.0568 ± 0.18257	9.0334 ± 0.31115	9.7733 ± 0.53686
	LPS	14.4014 ± 1.02473	14.3906 ± 0.49276	9.8023 ± 0.31115*	9.3018 ± 0.20602
GSH-Px (U/mg protein)	CON	15.5310 ± 1.45508	13.7160 ± 0.42498	13.5570 ± 0.60307	16.8665 ± 1.07325
	LPS	22.0999 ± 1.2071*	18.0544 ± 1.9736*	17.5424 ± 2.1281*	21.4042 ± 1.58232**
MDA (nmol/mg protein)	CON	0.3856 ± 0.02354	0.3444 ± 0.11768	0.2435 ± 0.08353	0.2268 ± 0.00967
	LPS	0.3634 ± 0.04299	0.3772 ± 0.07952	0.1684 ± 0.4000	0.2365 ± 0.04973

1 T-AOC – total antioxidant capacity; CAT – catalase; SOD – superoxide dismutase; GSH-Px – glutathione peroxidase; MDA – malondialdehyde; CON – control; LPS – lipopolysaccharide. Data represent the mean ± standard deviation (n = 6 in each group).

* – significant increase (P-value < 0.05);

** – highly significant increase (P-value < 0.01)

### Effect of LPS on the mRNA expression of thymic TLR4 pathway, related inflammatory factors and iNOS

**Fig. 2.**
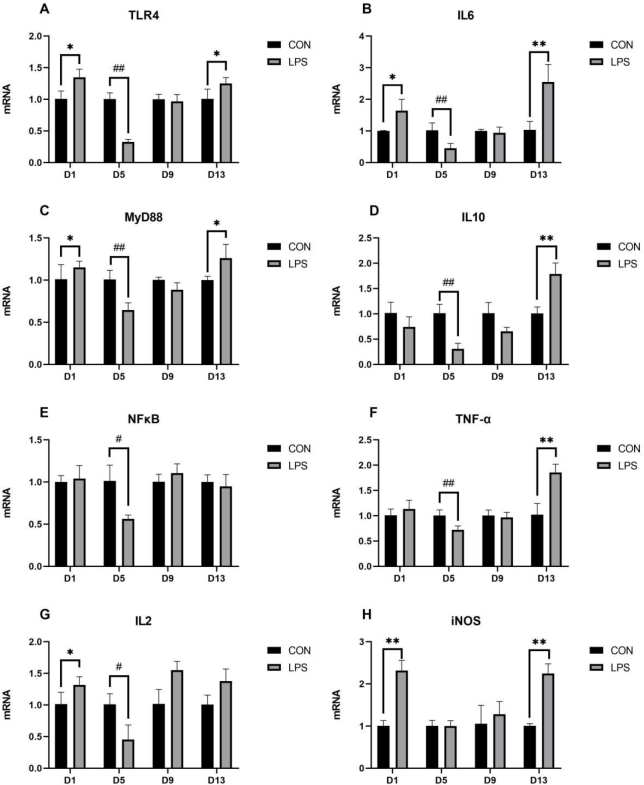
The mRNA expression of thymic toll-like receptor 4 (TLR4) pathway, related inflammatory factors and inducible nitric oxide synthase (iNOS). IL – interleukin; MyD88 – myeloid differentiation factor 88; NFκB – nuclear factor kappa B; TNF-α – tumour necrosis factor α; CON – control group; LPS – lipopolysaccharide group; * – significant increase (P-value < 0.05); ** – highly significant increase (P-value < 0.01); # – significant decrease (P-value < 0.05); ## – highly significant decrease (P-value < 0.01)

The relative expression of the thymic TLR4 pathway, associated inflammatory factors and iNOS mRNA is shown in Fig. 2. The TLR4 mRNA expression in the thymus of the LPS group was significantly increased on days 1 and 13 (P-value < 0.05) and highly significantly decreased on day 5 (P-value < 0.01). The MyD88 mRNA expression was significantly increased on days 1 and 13 (P-value < 0.05) but highly significantly decreased on day 5 (P-value < 0.01); NFκB mRNA expression was also significantly decreased on this day (P-value < 0.05). Compared with its expression in the CON group, IL-6 mRNA expression in the LPS group was significantly upregulated on day 1 (P-value < 0.05) and highly significantly upregulated on day 13 (P-value < 0.01), but was highly significantly downregulated on day 5 (P-value < 0.01). Interleukin-10 mRNA and TNF-α mRNA expression were highly significantly decreased on day 5 (P-value < 0.01) but highly significantly increased on day 13 (P-value < 0.01). The final interleukin, IL-2, was found to have its mRNA expression in the thymus of the LPS group significantly higher on day 1 (P-value < 0.05) but significantly lower on day 5 (P-value < 0.05). In addition, iNOS mRNA expression was highly significantly elevated on days 1 and 13 (P-value < 0.01).

### Effect of LPS on the mRNA expression of the thymic JAK-STAT and Keap1/Nrf2 pathways

**Fig. 3.**
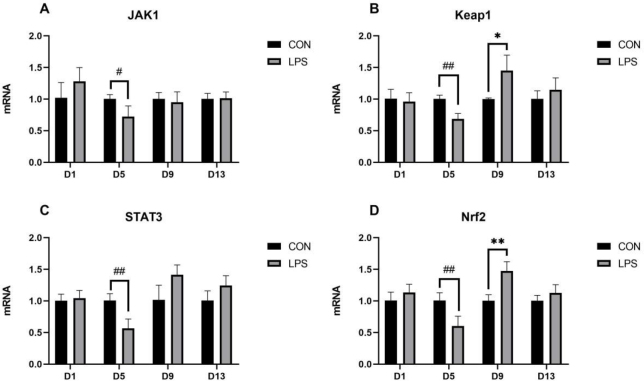
The mRNA expression of thymic JAK-STAT pathway and Keap1/Nrf2 pathway. A – janus kinase 1 (JAK1); B – kelch-like ECH-associated protein 1 (Keap1); C –signal transducer and activator of transcription 3 (STAT3); D – nuclear factor erythroid 2-related factor 2 (Nrf2). * – significant increase (P-value < 0.05); ** – highly significant increase (P-value < 0.01); # – significant decrease (P-value < 0.05); ## – highly significant decrease (P-value < 0.01)

The relative expression of thymic JAK-STAT and Keap1/Nrf2 pathway mRNA is shown in Fig. 3. Compared with its expression in the CON group, the expression of JAK1 mRNA in the LPS group was significantly decreased on day 5 (P-value < 0.05), as was the expression of STAT3 mRNA on the same day, but highly so (P-value < 0.01). The expression of Keap1 mRNA and Nrf2 mRNA in the thymus of the LPS group were both highly significantly decreased on day 5 (P-value < 0.01) but were significantly upregulated (Nrf2 highly significantly) on day 9 (P-value < 0.05 and P-value < 0.01, respectively).

## Discussion

The state of oxidative stress reflects the imbalance between oxidants (mainly oxygen radicals) and antioxidants produced within the organism. Excessive production of ROS results in oxidative modification of cellular macromolecules including DNA, lipids, proteins, *etc*. and in subsequent macromolecular damage, which in turn inflicts tissue damage. The excessive production of ROS may also directly inhibit the activity of antioxidant enzymes such as SOD and CAT or deplete peroxidative molecules such as GSH (23). Some studies have shown that LPS challenges can increase the concentration of ROS and damage the intestinal barrier to increase its permeability, and excessive LPS can also be transferred to the bloodstream to affect other tissues and organs (10). Bi *et al*. (6) successfully constructed an oxidative stress model for broiler chickens by using *Escherichia coli* LPS. Lipopolysaccharide can induce inflammation, growth inhibition and multiple diseases and even death (37).

Total antioxidant capacity reflects the overall level of enzymatic and non-enzymatic antioxidants *in vivo* (17). In the antioxidant system, superoxide is converted to hydrogen peroxide by SOD, which is then further degraded by CAT and GSH-Px, and when these antioxidants are elevated, it indicates that the body is fighting oxidative stress (30). Our results showed that CAT and SOD activities in the thymus were significantly higher in the prophase, probably because of the activation of immune function in this stage. The GSH-Px activity in the thymus increased throughout the experimental period. The unchanged T-AOC activity may be due to the decreased function of the non-enzymatic antioxidant system. Malondialdehyde is a lipid peroxide produced by the body, of which the level can serve as an indicator of the extent of oxidative damage. There was no significant change in MDA activity in this study, which may be attributed to its low expression in the thymus.

Toll-like receptor 4 is one of the more widely investigated factors in the toll-like receptor family. It can be activated by LPS and trigger a pro-inflammatory response that promotes the clearance of invading bacteria and induces tissue repair (33). In the context of LPS-induced TLR4 pro-inflammatory signalling, NFκB is continuously activated, controlling the production of pro-inflammatory factors such as TNF-α and IL-6 while stimulating the expression of interferon-induced chemokines such as IL-10. Tumour necrosis factor α is a typical pro-inflammatory factor and can promote vasodilation, increase vascular permeability and stimulate pain receptors, thus triggering typical inflammatory response manifestations such as redness, swelling, heat and pain. Interleukin 2 and IL-6 play important roles in regulating the proliferation, differentiation and function of immune cells (7). In a mouse model of acute atrophy of the thymus induced by LPS, the transcription level of IL-6 in thymic epithelial cells was elevated 38-fold (48). Initially designated as T-cell growth factor, IL-2, expressed correlatively with immune function, is the principal cytokine that stimulates T-cell proliferation. It is predominantly produced by CD4^+^ T helper cell 1 (16). Regulatory T cells inhibit the activation and proliferation of CD4^+^ T cells (9) and play a central role in suppressing the immune response by decreasing the expression of relevant inflammatory pathways and inflammatory factors and inhibiting T cell activity (24). Inducible NOS is an enzyme that catalyses the production of NO from L-arginine, and once activated, it catalyses the production of large amounts of NO. It is regulated by a variety of transcription pathways, *e.g*. those of NF-κB, JAK/STAT-1 and AP-1, and the pathways are dependent on the type of iNOS inducer (39, 51). Lipopolysaccharide or inflammatory factors such as TNF-α, IL-1 and IFN-γ first bind to cell surface receptors, activate kinases and induce intracellular proteins and specific transcription factors such as NF-κB and STAT-1a to translocate to the nucleus, where they bind to the promoter region of the *iNOS* gene and induce NO expression (8). In addition, NO production further promotes iNOS synthesis and expression.

As illustrated by expression results for thymic TLR4 signalling pathway–related factors and inflammatory factors, the mRNA expression of TLR4, MyD88, IL-6 and IL-2 in the thymus of piglets increased significantly on day 1 after LPS injection, which indicated that the thymus was immunologically activated immediately; however, the mRNA expression of these factors and NF-κB, TNF-α and IL-10 was significantly reduced on day 5, which is probably attributable to the thymic atrophy and immunosuppression caused by the overexpression of IL-6. Additionally, the mRNA levels of TLR4, MyD88, IL-6, TNF-α and IL-10 were significantly elevated on day 13, indicating that the inflammatory pathway was reactivated and the thymus immune-related functions were gradually restored. The expression of iNOS mRNA and NO content increased on days 1 and 13 after LPS induction, which indicated that the immune system was activated at the beginning, the thymus secreted a large amount of NO to resist inflammation and stress and was in the recovery stage in the middle period, and that inflammatory pathways and the antioxidant system were reactivated and the expression of iNOS mRNA and NO content was increased under continuous LPS induction.

The JAK/STAT pathway is a classic pathway of inflammation that can participate in the proliferation, differentiation, survival and apoptosis of immune cells and regulate the immune function and other processes (27). Lipopolysaccharide regulates IL-6 gene expression *via* NF-κB by inducing the TLR4 signalling pathway (50), and up or downregulation of IL-6 has the effect of increasing or decreasing the protein expression of the JAK/STAT signalling pathway (15). This allows this pathway to regulate the expression of downstream genes such as the NLRP3 inflammasome, thereby modulating the inflammatory response (52). In the present study, the mRNA expression of JAK1 and STAT3 in weaned piglets under LPS induction decreased significantly on day 5 and recovered in the later period, which was basically consistent with the changes of IL-6. However, the alterations in JAK1/STAT3 mRNA levels on days 1 and 13 did not align with those observed in IL-6 mRNA levels, which may be attributed to the minimal fluctuations in IL6 mRNA content. These suggest that this signalling pathway may play an important role in inflammatory response and immune regulation in LPS-induced piglets.

Oxidative stress and inflammation are two processes that are mutually reinforcing. An imbalance in the host’s antioxidant profile results in the release of inflammatory mediators, and conversely inflammatory mediators released during the inflammatory response can exacerbate oxidative stress. The Keap1/Nrf2 pathway is known as the most important endogenous antioxidant pathway because it regulates the transcription activity of ARE-dependent detoxification enzyme genes and antioxidant genes including CAT, SOD, GST, quinone oxidoreductase, *etc*. (42). In typical circumstances, Nrf2 and Keap1 bind into the form of an inactive dimer to maintain the oxidative system in a stable state. However, Nrf2 can dissociate from Keap1 when stimulated by external oxidative stressors or nucleophilic substances (4). Deficiency in Nrf2 leads to a sustained elevation of IL-1β and TNF-α in the inflammatory response; alternatively, Nrf2 can be viewed as another mode of cellular defence against oxidative stress which acts to regulate the expression of protease subunit genes in order to increase protease activity and further degrade oxidative stress-induced aberrant proteins (26). In this experiment, the mRNA levels of Keap1 and Nrf2 decreased on day 5, probably because the piglets’ thymus were in a state of immunosuppression. This resulted in the inhibition of the expression of the antioxidant pathway, which is consistent with the mRNA expression of the inflammatory pathway described above. On day 9, the mRNA expression of Keap1 and Nrf2 was elevated, indicating that an antioxidant response had been initiated once again. This is consistent with the trend of SOD, which is capable of responding rapidly to oxidative stress.

## Conclusion

In the LPS group, the CAT expression in the thymus was significantly elevated on days 1 and 5, the activity of SOD was significantly higher on day 9 and GSH-Px activity in the thymus was elevated through the four experimental periods. Lipopolysaccharide induced immune stress in the thymus of piglets in the prophase, which enhanced the mRNA expression of the TLR4 pathway and related inflammatory factors (IL-6 and IL-2) in the thymus and improved its immune ability. The thymus was immunosuppressed on day 5, which decreased the mRNA expression of the related inflammatory pathways (the TLR4 and JAK-STAT pathways), the antioxidant pathway (Keap1/Nrf2 pathway), and the related inflammatory factors (TNF-α, IL-10, IL-6, IL-2). However, sustained LPS administration caused the reactivation of these pathways, which increased the mRNA expression of the TLR4 and Keap1/Nrf2 pathways and some inflammatory factors (TNF-α, IL-10 and IL-6). Overall, lipopolysaccharide induction led to heightened activation of the thymic immune system in piglets during the prophase. However, this activation was accompanied by some atrophy and immunosuppression mid-experiment. Nevertheless, in the later period, the relevant inflammatory and antioxidant pathways were activated again, indicating a gradual recovery of immune function. This study provides more theoretical knowledge regarding the mechanisms of inflammation and stress in the immune system of weaned piglets in response to Gram-negative bacterial infections.
